# The prevalence of low back pain among female hospital staff at childbearing age

**DOI:** 10.7717/peerj.9199

**Published:** 2020-05-15

**Authors:** Fadi Al-Hadidi, Isam Bsisu, Bassem Haddad, Saif Aldeen AlRyalat, Mamoun Shaban, Nada Matani, Sondos Dehidi, Yasmeen Khater, Rana Shahrouri, Tasnim Al Muzayen, Hashem Al Hawamdeh

**Affiliations:** 1 Department of Special Surgery, School of Medicine, University of Jordan, Amman, Jordan; 2 Department of Anesthesia and Intensive Care, School of Medicine, University of Jordan, Amman, Jordan; 3 Department of General Surgery, School of Medicine, University of Jordan, Amman, Jordan; 4 Department of Pediatrics, Hamad Medical Corporation, Doha, Qatar; 5 School of Medicine, University of Jordan, Amman, Jordan

**Keywords:** Low back pain, Nursing, Female, Pregnancy, Hospital staff, Ergonomics

## Abstract

**Background:**

Low back pain (LBP) is considered the most common work-related musculoskeletal disorder among female healthcare workers. The aim of this study is to compare the prevalence of LBP and non-ergonomic risk factors between female nurses, office workers, and patient transporters, and the effect of pain on job performance.

**Methods:**

Based on semi-structured interviews, we conducted a cross-sectional study on Jordanian female hospital workers between January and July, 2017.

**Results:**

We included 209 participants with a mean age of 35.57 ± 8 years from four Jordanian medical centers. Nurses have significantly higher frequency of LBP (82.5%; *p* = 0.05) compared to both office workers (67.5%) and patient transporters (68.6%). The mean difference in pain score using Visual Analogue Scale (VAS) after treatment varied significantly (*p* = 0.003), since it was 28.2 (±35.4) for office workers, compared to 22.8 (±26.5) for nurses and 6.5 (±33.7) for patient transporters. A higher frequency of nurses reported that LBP affected their job performance (64.9%; *p* = 0.013), and 43.3% of them reported having previous sick leaves due to LBP (*p* = 0.008).

**Conclusions:**

LBP is common among female hospital workers, with significantly higher prevalence among female nurses when compared to other female hospital staff.

## Introduction

Low back pain (LBP) is a major healthcare issue with a high lifetime prevalence; Almost 80% of the world population experience LBP at some time during their lives (Andersson, 1999; Rubin, 2007). LBP is considered the most common work-related musculoskeletal disorder among healthcare workers (Serranheira, Sousa-Uva & Sousa-Uva, 2015; Smith et al., 2003; Trinkoff et al., 2003; Yan et al., 2017). Seeking healthcare services for LBP has increased over the past two decades (Freburger et al., 2009), and it was ranked as the fifth most common reason of consulting a physician (Altinel et al., 2008), increasing burden on healthcare system (Rapoport et al., 2004), as well as reducing productivity, lowering the quality of life, in addition to being the main reason for sick leaves and early retirement of hospital staff (Juniper, Le & Mladsi, 2009).

Females involved in the healthcare professions are at increased risk of developing LBP (Ghoussoub et al., 2016; Yan et al., 2017), which can be attributed to occupational risk factors like unhealthy postures such as bent or twisted postures, standing for long periods, heavy physical workload, repetitive movements, monotonous tasks, manual handling of heavy objects and transfer of patients (Nourollahi, Afshari & Dianat, 2018; Pheasant & Stubbs, 1992; Smedley et al., 1995). Several previous studies found correlations between LBP and low mood, stress, and job dissatisfaction, which will ultimately have a negative impact on the healthcare system by decreasing the productivity of healthcare workers (Bener et al., 2013; Smedley et al., 1997).

Each year, 211 million women get pregnant around the world (Van Lerberghe, 2005), of which 50% experience LBP (Katonis et al., 2011), which is considered to be the most common musculoskeletal complaint during pregnancy (Ritchie, 2003). In addition, it also continues to be a common complaint in the postpartum period and up to 11 years after pregnancy, lowering the quality of life for many women (Elden et al., 2016; Morino et al., 2017; Norén et al., 2002). LBP and lumbopelvic pain during pregnancy may result from mechanical, hormonal, or other factors associated with the changes in the body (Katonis et al., 2011) such as ligament laxity due to pregnancy-related hormones in early pregnancy (Morino et al., 2017; Vermani, Mittal & Weeks, 2010), or weight gain and a shift in the center of gravity in late pregnancy (Morino et al., 2017). Since most of female hospital workers in Jordan are in the childbearing age (Suliman, 2018), it is worth mentioning that the prevalence of LBP tends to increase in pregnancy (Casagrande et al., 2015).

The objective of this study is to compare the prevalence of LBP between different hospital staff workers, and compare them in term of non-ergonomic risk factors, in addition to comparing the effect of pain on their job performance.

## Materials and Methods

### Data collection and study design

Based on semi-structured interviews by medical students (Louise Barriball & While, 1994), we performed a cross-sectional study on female nurses, office workers, and patient transporters that were employed in Jordan University Hospital (JUH), King Hussein Cancer Center (KHCC), Al-Bashir Public Hospital, and Prince Hamzah Public Hospital, all of which are hospitals in Amman, the capital of the Hashemite Kingdom of Jordan. The sample of the study was chosen randomly using www.randomizer.org, 209 questionnaires were filled over the period of 6 months between January and July, 2017.

### Interview questions

The interview questions included the age of participant, level of education, weight before pregnancy and current weight, height, Body Mass Index (BMI), previous pregnancies, parity, gestational age if currently pregnant, history of previous twin pregnancies, smoking habits, whether the drive cars or not, time spent watching TV as well as time spent on internet, having assistance at home and exercises (type and duration). Regarding occupational information, inquiries were made about the job title, working hours and job satisfaction. The final part of the questionnaire inquired about the current or previous history of LBP. If present, more information was obtained regarding the duration of pain in years, seeking treatment, type of current or past treatments, and the severity of back pain before and after pregnancy using Visual Analogue Scale (VAS); where the participants were asked to rate their back pain on a horizontal scale from 0 to 100, where 0 represents no pain and 100 represents intolerable pain (1). In addition, the number and duration of sick leaves due to back pain were also obtained from the study participants.

### Inclusion and exclusion criteria

We included pregnant and previously-pregnant women between the ages of 22 and 60 years who work at JUH, KHCC, Al-Bashir Public Hospital and Prince Hamzah Public Hospital with one of the following job titles: nurse, office worker, or patient transporter. Any staff member with other job titles were not included in the study. Male hospital workers with the same job titles were not included, neither were staff members who had previous back surgeries or have been previously diagnosed with spine disorders, deformities and diseases.

### Ethical approval

The study was approved by Institutional Review Board (IRB) at JUH (approval number: 67/2018/4231). An oral consent was obtained at the beginning of the interview after explaining the primary aims of the study and the confidentiality of obtained information. No identifying personal data were requested during the interview, and the collected data was used solely for statistical analysis.

### Statistical analysis

SPSS 21.0 (Chicago, IL, USA) was used to analyze the data. The mean value (±standard deviation) was used to describe age, median (interquartile) to describe number of previous pregnancies and count (frequency) to describe work.

We adopted a model building strategy for pain-related factor assessment, where we performed chi-square test for previous pregnancy, parity, smoking, car driving, exercise, level of education, and work and we performed One-way ANOVA and post-hoc analysis of the least significant difference (LSD) for age, BMI, duration on TV, duration on internet, and difference in VAS pain score. We tested assumptions relevant for each test. A *p*-value of 0.05 was used as the significant threshold. Any factor with a *p*-value < 0.05 from the previous tests will be entered to the logistic regression analysis, where the *p*-value threshold was 0.05. Moreover, chi-square test and independent sample *t*-test were used to compare between participants who were currently pregnant and non-pregnant female hospital workers where the significant threshold was a *p*-value < 0.05.

## Results

A total of 209 women were included in this study with a mean age of 35.57 years (±7.99). They were 97 (46.4%) nurses, 77 (36.8%) office workers and 35 (16.7%) patient transporters. A total of 30 (14.4%) participants were pregnant at the time of the interview, while the number of participants who were not currently pregnant, but has been pregnant in the past, was 179 (85.6%). The median work duration was 8 h, and did not differ significantly between the three groups. Table 1 provide detailed characteristics for each type of work.

**Table 1:** Comparison between nurses, office workers, and porters. A comparison between nurses, office workers, and porters in demographic factors as well as the characteristics and severity of their low back pain.

	Nurses	Office workers	Patient transporters	*p*-Value
Mean age (years)	33.8 (±6.96)	36.3 (±8.41)	39 (±8.59)	0.002
Mean BMI (Kg/m^2^)	24.1 (±3.88)	23.3 (±3.24)	25.7 (±4.82)	0.009
Median number of pregnancies	3 (2–5)	3 (2–4)	4 (3–5)	0.24
Twins	6.2%	7.9%	11.4%	0.606
Smoking	5.2%	15.6%	20%	0.022
Car driving	34%	66.2%	14.3%	<0.001
Assistance at home	11.3%	20.8%	2.9%	0.026
Exercise	21.6%	41.6%	37.1%	0.014
Satisfaction	70.1%	63.6%	65.7%	0.656
Back pain	82.5%	67.5%	68.6%	0.053
Mean duration of back pain (years)	4.7 (±4.7)	8.3 (±9.5)	6.2 (±7.3)	0.017
Seeking treatment	55.2%	42.9%	40%	0.18
On treatment	50%	46.8%	54.3%	0.76
Pain affecting work	64.9%	42.9%	50%	0.013
Sick leaves	43.3%	27.3%	17.1%	0.008

**Note:**

BMI: body mass index.

Upon comparing type of work with back pain, significant differences (*p* = 0.05) were found, as nurses having a higher frequency of pain (82.5%) compared to both office workers (67.5%) and patient transporters (68.6%). The mean duration for LBP was 6.1 ± 7.1 years. Upon comparing between the three groups in terms of the duration of LBP, we found a significant difference between the studied groups (*p* = 0.017) (Table 1). Post-hoc analysis showed that nurses had significant less duration of LBP compared to office workers (*p* = 0.004), while no significant difference was found between nurses and patient transporters, nor between office workers and patient transporters.

Upon analyzing the difference of VAS-pain score after treatment and type of work, significant difference (*p* = 0.003) was found; the mean difference in pain score after treatment for office workers was 28.2 (±35.4), for nurses was 22.8 (±26.5), and for patient transporters was 6.5 (±33.7).

No significant difference could be found between the median number of previous pregnancy and having back pain. No significant differences were found in the frequency of back pain and having twins

The following variables had a *p* value < 0.5 and were included in the logistic regression analysis: Age (0.002), smoking (0.022), car driving (<0.001), exercise (0.014) and BMI (0.009) (Table 1). On the regression analysis, the overall model was significant at 0.036, but only BMI had a significant *p* value (0.020) from the included variables, with an odds ratio of 1.154 (Table 2).

**Table 2:** Logistic regression analysis for the main studied risk factors of LBP. The overall model of the logistic regression analysis was significant at 0.036, but only BMI had a significant *p* value (0.020) from the included variables, with an odds ratio of 1.154.

		B	S.E.	Wald test	df	*p*-Value	OR
Step 1^a,b^	Age	0.040	0.029	1.870	1	0.171	1.040
	BMI	0.143	0.061	5.419	1	0.020	1.154
	Smoking	0.321	0.695	0.213	1	0.644	1.378
	Car driving	−0.360	0.419	0.738	1	0.390	0.697
	Time watching television	0.212	0.184	1.328	1	0.249	1.236
	Time on internet	0.006	0.092	0.005	1	0.945	1.006
	Exercise	−0.311	0.414	0.566	1	0.452	0.733
	Constant	−3.354	1.576	4.527	1	0.033	0.035

**Notes:**

a Variables entered on step 1, age, BMI, smoking, car driving, time watching television, time spent on internet, and exercise.

b B, unstandardized beta; S.E, standard error; df, degrees of freedom; OR, odds ratios.

Of the 30 (14.4%) participants who were pregnant at time of the investigation, 23 (76.7%) had LBP, compared to 133 (74.3%) in female hospital workers who were not currently pregnant (*p* = 0.783). Neither the number of participants who sought medical treatment (*p* = 0.086), nor the number of participants on treatment (*p* = 0.059) were significantly different between pregnant and non-pregnant participants. However, the mean duration of LBP was significantly different between them, with a mean duration of 2.1 ± 2.2 years for currently pregnant participants, compared to 6.8 ± 7.5 years for those who were not currently pregnant (*p* < 0.001). We further investigated their job satisfaction, which did not show a significant difference between pregnant and non-pregnant workers (*p* = 0.704).

## Discussion

In this article, the prevalence of LBP and its relationship with pregnancy in female hospital staff was investigated. The prevalence of pregnancy-related LBP and pelvic girdle pain PGP varied wildly between studies, with their prevalence ranging between 3.90% and 89.88% (Kovacs et al., 2012; Wu et al., 2004; Bastiaanssen et al., 2005). It has been demonstrated that about 45% of pregnant women and 25% of postpartum women (from birth till 6 to 8 weeks after birth (Leahy-Warren & McCarthy, 2011)) suffer from either lumbar pain or pelvic girdle pain (Larsen et al., 2013; Sencan et al., 2018). According to a previous study done on nurses from both genders in Jordan, the prevalence of LBP was 69%, 1-year prevalence was 78.9%, while the cumulative prevalence was 83.6%, most of them were females (70.3%) (Suliman, 2018). In Tunisia, LBP cumulative life-time prevalence among hospital staff was 57.1%, while LBP yearly prevalence was 50.1% and LBP incidence rate was 3.14%, without the presence of any interrelationship between professional categories and LBP (Bejia et al., 2005). Among hospital staff in a university hospital in Lebanon, the prevalence of LBP was 54% with the strongest risk factor being female gender (Ghoussoub et al., 2016), while another study showed that 44.8 % of Lebanese office workers suffer from back pain, which was also more prevalent among female gender (Bawab et al., 2015).

Previous literature revealed that administrative staff and workers had higher prevalence of LBP than nurses, which was justified by the prolonged sitting position and the sedentary nature of the activities of administrative staff, while it is explained by the conditions of work and heavy loads handling for workers and porters (Bejia et al., 2005; Massironi et al., 1999; Troussier et al., 1993; Caillard et al., 1987; Czernichow & Doucet, 1987; Bordes, Oliva & Fortin, 1996). On the contrary, in the present study which was conducted exclusively on female hospital staff, nurses had significantly higher frequency of pain (82.5%) compared to both office workers (67.5%) and patient transporters (68.6%). Although nurses were the group with the lowest mean age, the group who was assisted most at home, and despite that they were more satisfied with their job. It was also found that their jobs as nurses was more negatively affected by their pain (64.9%), more than office workers (42.9%) and patient transporters (50%). These findings raised the question: Is nursing as a job an independent risk factor for developing LBP in pregnant women? Further studies are highly recommended to investigate the relationship between the occupational and ergonomic risk factors of each hospital staff job and its relationship with LBP, since the different prevalence of LBP between different workers might be related to the nature of their job, which differs from an institution to another.

Recent studies have shown that the risk factors for developing LBP among hospital staff can be divided onto individual, professional (ergonomic), physical, psychosocial and lifestyle factors (Sorour & El-Maksoud, 2012; Harcombe et al., 2010; Carugno et al., 2012). Suliman (2018) found that the factors associated with LBP in Jordanian nurses other than the female gender were older age, being overweight, and having longer experience in nursing. Moreover, Bejia et al. (2005) found that the factors associated with LBP among hospital staff were age, high BMI, being married or divorced, smoking, past medical history of LBP, extra professional activity, migraine, years of service and weight lifting, while exercise was a protective factor against LBP. In a study done on pregnant women in Iceland, Lindal et al. (2000) concluded that smokers had LBP more frequently during and after pregnancy, while previous births and birth weight were not found to correlate positively with LBP. In the present study, none of the previously mentioned risk factors were found to be significantly associated with LBP among female hospital staff. In addition, neither the number of previous pregnancies, nor the presence of twin pregnancies were associated with LBP, which was consistent with the previous studies (Bejia et al., 2005; Suliman, 2018).

Patient-reported outcome measures are increasingly becoming the standard of care throughout the orthopedic community (Ayers, 2017; Al-Hadidi et al., 2019). The mean difference in VAS scores after treatment for office workers was 28.2 (±35.4), compared to 22.8 (±26.5) for nurses and 6.5 (±33.7) for patient transporters. The clinically significant decrease in the severity of LBP is 50% reduction in pain intensity, and it corresponds to the patient impression of improvement (Rowbotham, 2001). Further researches are encouraged to investigate the role of medical treatment, rehabilitation, and lifestyle modification in decreasing the severity of LBP in the setting of healthcare workers and hospital staff, in addition to the role of healthy practice guidance in decreasing the incidence of LBP.

The main limitation of this study is that it did not compare LBP in the study objects at specific timing in the post-partum period for previously pregnant women and intrapartum period for currently pregnant women, which we recommend it to be investigated in future studies in order to determine the LBP severity at different maternity periods. In addition, the correlation between LBP and the time since last pregnancy was not studied in previously pregnant women. Second, this study did not compare between the occupational risk factors, such as workstation designs and office temperature, which may be of great importance in taking prophylactic measures to prevent LBP, especially that recent studies suggested that women have a higher prevalence of LBP and are more susceptible to environmental risk factors than men (Ye et al., 2017). Third, psychosocial information about the participants was not included in the study. Moreover, comparison was not done between different treatment modalities in the effectiveness in decreasing LBP severity, for which further studies should compare between medical treatment, rehabilitation, and interventional treatment in order to be able to come up with the most efficacious treatment for LBP in working women in childbearing age.

## Conclusions

In conclusion, LBP is a common cause of disability among female hospital staff, with significantly higher prevalence among female nurses when compared to other female hospital staff. Being at high risk for LBP, nurses should be aware of the importance of occupational and non-occupational risk factors in order to decrease their risk of developing LBP. This will ultimately lead to better healthcare delivery, better productivity and higher levels of job satisfaction.

## Supplemental Information

10.7717/peerj.9199/supp-1 Supplemental Information 1 Prevalence of low back pain in female hospital staff, taking into consideration non-ergonomic risk factors. Low back pain is common among female hospital workers, with significantly higher prevalence among female nurses when compared to other female hospital staff. SPSS 21.0 (Chicago, IL, USA) software was used for statistical analysis.

## Additional Information and Declarations

### Data Availability

The following information was supplied regarding data availability: The research data is available in the Supplemental File.
